# Gut microbiome-derived hydrolases—an underrated target of natural product metabolism

**DOI:** 10.3389/fcimb.2024.1392249

**Published:** 2024-06-10

**Authors:** Jiaxin He, Xiaofeng Liu, Junming Zhang, Rong Wang, Xinyuan Cao, Ge Liu

**Affiliations:** ^1^ People’s Hospital of Ningxia Hui Autonomous Region, Pharmacy Department, Yinchuan, China; ^2^ School of Pharmacy, Lanzhou University, Lanzhou, China; ^3^ Ningxia Medical University, School of Basic Medicine, Yinchuan, China

**Keywords:** natural product, microorganisms, gut microbiome-derived hydrolases, secondary metabolism, mechanism of enzymatic deconstruction

## Abstract

In recent years, there has been increasing interest in studying gut microbiome-derived hydrolases in relation to oral drug metabolism, particularly focusing on natural product drugs. Despite the significance of natural product drugs in the field of oral medications, there is a lack of research on the regulatory interplay between gut microbiome-derived hydrolases and these drugs. This review delves into the interaction between intestinal microbiome-derived hydrolases and natural product drugs metabolism from three key perspectives. Firstly, it examines the impact of glycoside hydrolases, amide hydrolases, carboxylesterase, bile salt hydrolases, and epoxide hydrolase on the structure of natural products. Secondly, it explores how natural product drugs influence microbiome-derived hydrolases. Lastly, it analyzes the impact of interactions between hydrolases and natural products on disease development and the challenges in developing microbial-derived enzymes. The overarching goal of this review is to lay a solid theoretical foundation for the advancement of research and development in new natural product drugs and personalized treatment.

## Graphical Abstract

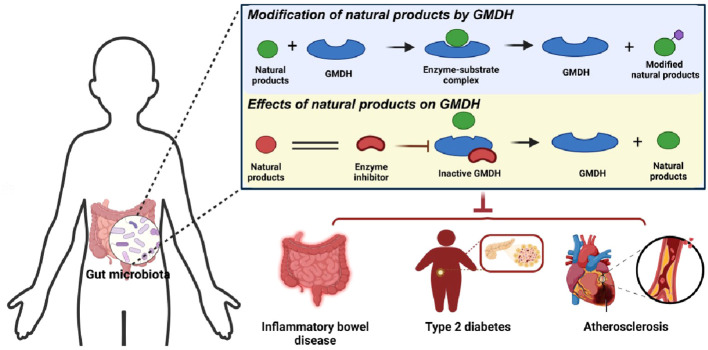

Gut microbiome derived hydrolases directly or indirectly influence the metabolic processes of natural products, thereby impacting the occurrence and development of diseases.

## 1 Introduction

The gut microbiota has a significant impact on the modification of oral drugs. Once oral drugs enter aims intestine, they undergo various modifications by microbiome-derived enzymes (MDE) such as hydrolases, lyases, oxidoreductases, and transferases (Xie Y. et al., 2020). These modifications alter the effectiveness and toxicity of the drugs (Zhang et al., 2018; Weersma et al., 2020). Among these, hydrolases, such as glycoside hydrolases, carboxylesterases, amide hydrolases, and bile saline hydrolases, play a significant role in drug metabolism (Tomioka et al., 2022). What’s more, It has been reported that drugs metabolized by hydrolases make up more than 30% of drugs co-metabolized by microorganisms (Zimmermann et al., 2019). Natural products are a vital component of pharmaceuticals. Over the past 40 years, 1,881 new drugs have been approved globally, with approximately 23.5% originating from natural products and their derivatives. This includes 71 natural products, accounting for 3.8%, and 356 natural product derivatives, accounting for 18.9% (Newman and Cragg, 2020). However, natural product drugs are primarily administered orally, exhibiting pharmacological activity only after microbial transformation or metabolism into more potent secondary glycosides or aglycones (Lai et al., 2010; Zhou et al., 2019). A notable example is sulforaphane, which is converted to sulforaphane isothiocyanate by hydrolase in the cecum (Lai et al., 2010; Sikorska-Zimny and Beneduce, 2021; Yuanfeng et al., 2021). And the hydrolysis of Malonyl isoflavone glucosides in the cecum produces aglycones (Yonemoto-Yano et al., 2014). In addition, it has recently been reported that dipeptidyl peptidase 4 (DPP4), a hydrolase derived from microorganisms, can collaboratively degrade in glucagon-like peptide-1 (GLP-1) *in vivo*, thereby influencing the metabolism of natural product drugs (Wang K. et al., 2023). This underscores the importance of investigating the metabolic influence of gut microbiome-derived hydrolases (GMDH) on natural products.

## 2 Regulation of natural products by gut microbiome-derived hydrolases

As a superorganism composed of 10 to 10 trillion individuals, the gut microbiome possesses a gene set that is roughly 150 times larger than the human gene set (Levan et al., 2019; Nishida et al., 2022). It is estimated that the human gut microbiome alone harbors around 1000-1500 different species, many of which remain unidentified (Qin et al., 2010). Recent advancements in genomics technology have sparked increased interest among scientific researchers in exploring the role of intestinal microorganisms and their encoded enzymes. These gut microbiota in human intestinal metabolism by providing enzymes that are not produced by the human body. These enzymes are essential for breaking down complex polysaccharides, metabolizing drugs, and carrying out various metabolic functions (Rakoff-Nahoum et al., 2016; Zhao et al., 2022; Raba and Luis, 2023). Glycoside hydrolases, amid hydrolases, carboxylic esterase, bile saline hydrolases, and epoxide hydrolases produced by microorganisms directly change the specific structures of these natural products. The following article takes these five GMDHs as examples to describe the metabolic regulation of natural products by gut microbiome-derived hydrolases.

### 2.1 Glycoside hydrolase

**Figure 1:**
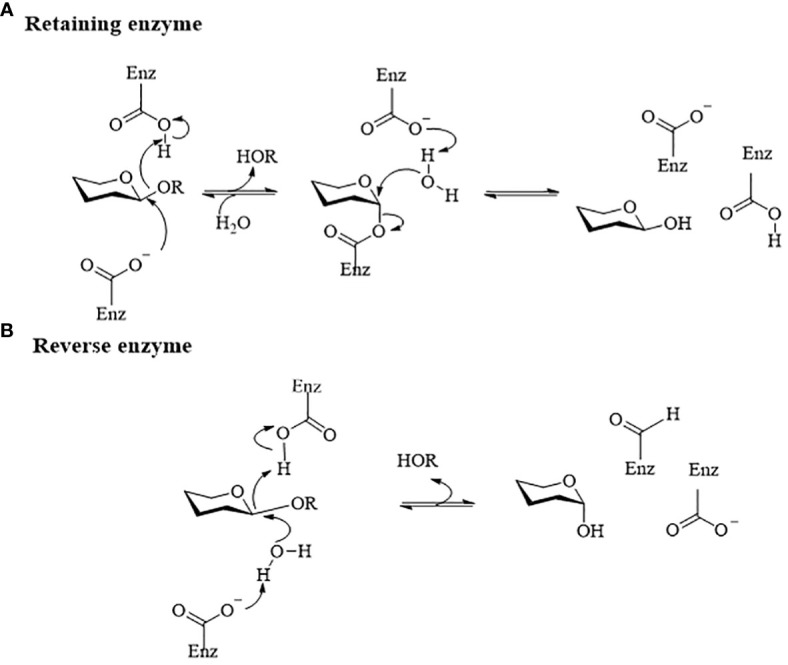
Diagram of the catalytic mechanism of glucoside hydrolase (Honda and Kitaoka, 2006). **(A)** Retention catalytic mechanism. Their catalytic mechanisms typically involve a ‘twostep’ process. In the first step, a carboxyl anion acts as a nucleophile and attacks the anomeric carbon on the glycosidic bond. Following the formation and breaking of bonds, the anomeric carbon configuration of the glycosyl molecule changes, forming an ester bond with the nucleophilic carboxyl group to produce a glycosylenzyme covalent intermediate and release a glycolipid molecule. In the second step, the active hydroxyl hydrogen of the glycosyl acceptor molecule interacts with the dissociated generalized acidbase pair carboxyl ion. This leads to the formation of an oxygencontaining carbocation-like transition state at the anomeric carbon, resulting in a second flip of the anomeric carbon configuration and the formation of a covalent bond with the acceptor hydroxyl oxygen to complete the reaction. **(B)** Reverting enzyme mechanism. The hydron molecule attacks the anomeric carbon from the opposite direction without a second flip of the configuration, leading to the formation of products with different configurations.

Gut microbiome-derived glycoside hydrolases catalyze the hydrolysis of glycosidic bonds in glycoside natural products to produce hemiacetal and corresponding free glycoside ligands (Xu et al., 2023). These enzymes play a crucial role in altering the structure and bioavailability of glycosides, which are commonly found in natural drugs and considered ‘prodrugs’ in pharmacokinetics (Zhou et al., 2022). Interestingly, the process of gut microbiome hydrolyzing glycoside compounds is mutually beneficial. Glycoside hydrolases from gut microbiota break down glycosidic bonds in glycosides using dicarboxylic acid residues and water molecules, releasing free glycosides and glycogens. These free glycosides can serve as energy sources to support the growth of specific microorganisms in the gut (Braune and Blaut, 2016). Glycoside hydrolases commonly catalyze hydrolysis reactions using a retention mechanism, as shown in **Figure 1A**. However, there are cases where hydrolysis reactions take place through inversion catalysis, as shown in **Figure 1B**. In inverting glucosidases, the acceptor molecule attacks the anomeric carbon from the opposite direction without a second flip of the configuration, leading to the formation of products with different configurations (Coines et al., 2019). Gut microbiome-derived glycosidases, based on their substrate specificity and catalytic mechanism, can be categorized as β-D-glucosidase, β-xylosidase, β-galactosidase, α-L-mannosidase, sialidase, among others. **Table 1** presents a summary of natural product drug hydrolysis by gut microbiome-derived glycosidases and their respective catalytic mechanisms, aiming to elucidate the involvement of endogenous hydrolases from the intestinal microbiome in the *in vivo* modification of natural product drugs.

**Table 1:** Metabolism of natural product drugs by glycoside hydrolases.

Hydrolytic enzyme category	Participating microbiota	Substrate	metabolic pathways	End-product	Ref.
β-galactosidase	*Sulfolobus sp*	stevioside (St)	classic	steviol	(Wan and Xia, 2015)
	*Bacillus megaterium YZ08*	naringin, polydatin, aesculin, and bergenin	–	–	(Zhou et al., 2017)
	*Penicillium sp*	1-*O*-acetyl-β-d-galactopyranose	retain	d-Galactose	(Zinin et al., 2002)
	*Anemarrhena asphodeloides Bunge*	timosaponin AIII (TA3)	reverse	M1	(Jia et al., 2014)
α-L-rhamnosidase	*Bifidobacterium breve, Fusobacterium K-60*	Rutin	classic	isoquercitrin, quercetin	(Zhang R. et al., 2015)
	*Rha78s*	Rutin and naringin	classic	Przewalskil	(Li et al., 2019)
	*B.thetaiotaomicron VPI-5482*	Epimedin A, B, C	retain	icariin A, B	(Wu et al., 2018)
	*Bifidobacterium catenulatum and Bifidobacterium pseudocatenultum*	Hesperidin	classic	Hesperetin	(Mas-Capdevila et al., 2020)
	*NA*	Ardipusillosides I	retain	Deglycosylated product	(Cao et al., 2015)
β-glucosidase	*Burkholderia GE 17-7*	ginsenoside Rb1		ginsenoside Rg3	(Fu et al., 2017)
	*Arthrinium* sp. *GE 17-18*			ginsenoside C-K	(Fu et al., 2016)
	*Escherichia coli*			ginsenoside Rg3 g2 Rh1	(Shin et al., 2015)
	*E. coli HGU-3*	Baicalin	classic	Baicalein;	(Han et al., 2016)
	*E. cellulosolvens ATCC 43171T*	Luteolin 7-O-glucoside;	retain	Luteolin;	(Braune and Blaut, 2012)
	*Bacteroides JY-6;*	Rutin	reverse	Quercetin-3-O-glucoside;	(Ferreira-Lazarte et al., 2021)
	*Escherichia* sp. *23*	Isorhamnetin-3-O-neohesperidoside		Isorhamnetin-3-O-glucoside;	(Du et al., 2017)
	*B.animalis subsp.* *Lactis AD011*	Quercetin 3-*O*-glucoside	retain	Quercetin;	(Youn et al., 2012)
	*Lactobacillus paracasei A221*	Kaempferol-3-*O*-sophoroside	retain	Kaempferol	(Shimojo et al., 2018)
	*Enterococcus.* sp. *8B, 8-2,9-2*	Astilbin	reverse	Taxifolin	(Zhao et al., 2021)
	*B. pseudocatenulatum*	Hesperidin	classic	Hesperetin	(Mas-Capdevila et al., 2020)
	*E. ramulus;*	Daidzin	classic	Daidzein	(Mace et al., 2019)
	*B. fragilis;*	Paeoniflorin	–	PM-I;	(He et al., 2007)
	*Bifidobacterium* sp. *strain SEN*	sennoside A and B	retain	Sennidin A/B-8-monoglucoside	(Matsumoto et al., 2012)
β-xylosidase	*Thermosaccharolyticum*	ginsenoside R1 R2	retain	ginsenosides Rg1 Rh	(Shin et al., 2014)
	*Dictyoglomus turgidum*	Epimedium B	reverse	Baohuoside I	(Tong et al., 2021)

The study demonstrates that steric hindrance plays a significant role in the hydrolysis of specific flavonoids by glycosidases in the process of intestinal microbial fermentation. While there is a considerable body of research on β-D-glucosidases, there is a noticeable gap in the literature regarding the impact of other glycosidases on natural product drugs. Therefore, further investigation is warranted to delve into the alteration of natural product drugs by various types of glycosidases, in order to comprehensively grasp the mechanism by which intestinal microorganisms act on natural product drugs and lay the groundwork for personalized treatment.

### 2.2 Amidohydrolase

**Figure 2:**
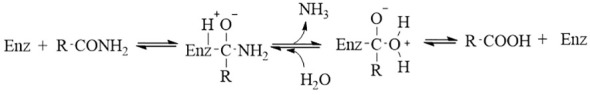
The catalysis mechanism of microbiome-derived amidohydrolase (Wu et al., 2020). Initially, the carbonyl amide forms an enzyme-substrate-acyl tetrahedral intermediate with the enzyme, followed by the rapid release of ammonia, resulting in the formation of an acyl-enzyme complex. Subsequently, the enzyme dissociates, leading to the production of the corresponding acid.

Amide bonds are a prevalent structural feature in numerous natural product drugs, with approximately 25% of them containing at least one amide bond (Jamalifard et al., 2019). Therefore, explaining the interaction between microbiome-derived amide hydrolase and natural product drugs is important for the development of new natural product drugs (Narendar Reddy et al., 2019). The catalytic mechanism of amidohydrolase is illustrated in **Figure 2**. However, the catalytic pathways involved in the metabolism of natural products by the body’s gut microbiota require further investigation (Wu et al., 2020). The amidohydrolase from *Escherichia coli YqfB* is currently the smallest monomeric amide hydrolase known, with catalytic activity towards N4-acylated cytosines found in natural products (Stanislauskienė et al., 2020). Common microbial-derived amide hydrolases are categorized into lactam hydrolases, ureases, fatty acid amide hydrolases, and broad-spectrum substrate amidases based on their respective substrates. Among these, lactam hydrolases have been extensively studied and documented (Bush, 2018). The impact of antibiotic natural product drugs on intestinal microorganisms is substantial. Following antibiotic treatment, there is a significant reduction in the species and abundance of the gastrointestinal microbiome (Maier et al., 2021). Consequently, certain intestinal microorganisms have developed amidohydrolases as a defense mechanism to degrade antibiotics and develop resistance. For instance, *Pseudomonas* cereus exhibits antibiotic resistance by utilizing endogenous amide hydrolase to catalyze the side chains D-phenylglycine and D-p-hydroxyphenyl glycine of β-lactam antibiotics (Pang et al., 2019; Camiade et al., 2020). Interestingly, previous perceptions of amide hydrolase produced by gut microbiota as detrimental to amide antibiotics have shifted. Recent studies indicate that introducing engineered bacteria that produce amidohydrolase in the intestine can reduce the likelihood of intestinal microorganisms developing antibiotic resistance post-treatment (Cubillos-Ruiz et al., 2022). Furthermore, urease, an amidohydrolase enzyme involved in the body’s urea cycle, seems to possess similar properties. *Helicobacter pylori*, for instance, sustains its colonization in the gastric mucosa by secreting urease (Fischbach and Malfertheiner, 2018). However, recent studies suggest that the proliferation of certain urease-positive bacteria like *Streptococcus thermophilus* and *Streptococcus salivarius* plays a crucial role in maintaining the balance of endogenous ammonia molecules in the body (Ni et al., 2017; Wang P. et al., 2023).

### 2.3 Carboxylesterase

Carboxylesterase (CES) are widely distributed in various tissues and organs of the body and are highly expressed in the liver, kidney, and small intestine (Hughey and Crawford, 2019). Historically, the lack of identified endogenous substrates led to the belief that the sole role of carboxylesterases was to shield cells from lipid bond-containing compounds (Hatfield et al., 2016). It is hypothesized that the hydrolysis of lipid natural products by carboxylesterase enzymes derived from intestinal microorganisms could serve as a mechanism to shield bacteria from external xenobiotics. The catalytic mechanism of CES primarily involves attacking the oxygen electrophile of the carbonyl carbon in the substrate with the help of serine residues. This forms an acylated enzyme intermediate, which is subsequently hydrolyzed to generate a new active product following the rearrangement of the carbonyl group. CES is particularly important in the microbial metabolism of natural products that contain lipid bonds in their molecular structure (Redinbo and Potter, 2005). Zhao’s study demonstrated that microbiome-derived CES catalyzes the conversion of albiflorin, an antidepressant natural product, to benzoic acid, which has a higher likelihood of crossing the blood-brain barrier and acting within the central nervous system (Zhao et al., 2018). Jin Yu and Ran Peng observed similar findings in their study on the microbial metabolism of paeoniflorin. They found that CES from *Bifidobacterium* can facilitate the conversion of paeoniflorin to benzoic acid (Yu et al., 2019; Peng et al., 2022). This indicates that CES from gut microorganisms may have a significant impact on the metabolism of natural antidepressants containing benzoic acid structures. Intestinal microorganism-derived CES plays a crucial role in the metabolism of lignoside by hydrolyzing it into salidroside and tyrosol (Yu H. et al., 2023). This enzyme also facilitates the conversion of certain natural product drugs with toxic effects into non-toxic products. For instance, diester diterpenoid alkaloids are transformed into less toxic monoester diterpene alkaloids through the hydrolysis of the C-8 and C-14 ester bonds (Zhang M. et al., 2015). Research conducted by Ramya revealed that CES derived from *B. cereus KC985225* in *P.xylosida* can enhance indoxacarb degradation efficiency by up to 20% (Ramya et al., 2016). Therefore, the structural design of lipid-based natural product insecticides may need to be modified to prevent degradation by carboxylesterases in the insect gut. In summary, microbiome CES plays a crucial role in mitigating the toxicity of natural product drugs. Emphasizing their catalytic effects on natural product drugs is essential for laying the groundwork for the development of novel natural product drugs.

### 2.4 Bile salt hydrolase

**Figure 3:**
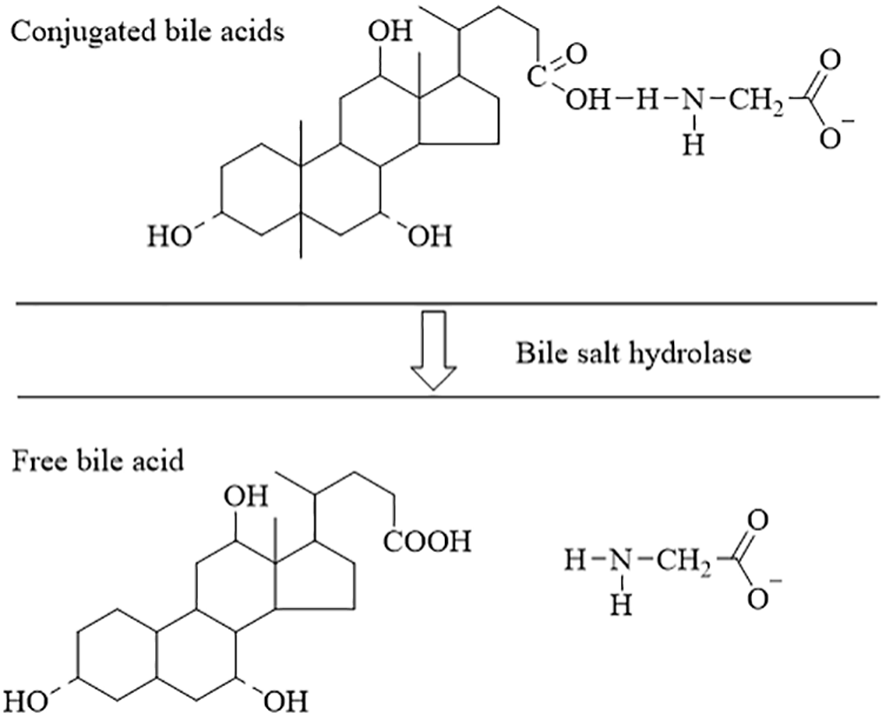
The process of dissociation of bile acids (Fiorucci and Distrutti, 2015; Ruiz et al., 2021; Wang et al., 2021). The conjugated bile acids are hydrolyzed to free bile acids by the action of the BSH.

Bile salt hydrolase (BSH) is a common enzyme found in the gastrointestinal tract of mammals. It is produced intracellularly by intestinal microbiota during growth and reproduction (Foley et al., 2023). The production of BSH not only assists intestinal microorganisms in dealing with high concentrations of bile salts in the intestine, but also supports the colonization and adhesion of certain beneficial bacteria (Joyce et al., 2014) The majority of gut microbiota have been found to exhibit BSH activity, including *Lactobacillus plantarum, Bifidobacterium sp, Bacteroides fragilis, Bacteroides vulgatus, Clostridium perfringens, Listeria monocytogenes, Lactobacillus* and *Bifidobacteria* (Ruiz et al., 2021, Fiorucci and Distrutti, 2015; Wang et al., 2021). As illustrated in **Figure 3**, BSH primarily catalyzes the hydrolysis of endogenous steroidal natural product BAs, releasing bound BAs in the intestinal bile acid pool into free bile acids and amino acid residues (Rimal et al., 2024). Numerous studies have highlighted the significance of BSH-mediated unbinding in BAs metabolism, impacting cholesterol synthesis, lipid metabolism, and glucose metabolism (Shen et al., 2021; Zhu et al., 2022). Recent research has also revealed that BSH enzymes are involved in degrading antibiotics, in addition to their role in BAs unbinding. For instance, Hiroyuki discovered that a BSH enzyme derived from *Lactobacillus paracasei JCM 5343T* not only shows resistance to bile salt stress but also mitigates the toxicity of β-lactam antibiotics to a certain extent (Kusada et al., 2022). In summary, In addition to BAs metabolism, there are numerous unexplored facets regarding the catalytic impact of gut microbiome-derived BSH on natural product drugs, including their response to antibiotic stress, which warrants further investigation.

### 2.5 Epoxide hydrolases

**Figure 4:**
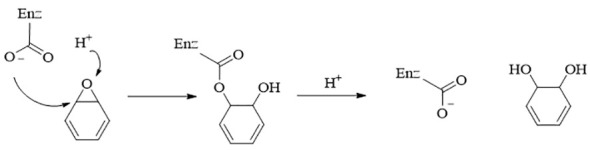
The catalysis mechanism of EHs. EHs form an enzyme-substrate ester-type complex with epoxide with the help of amino acid residues, which hydrolyzes to form diols (Chen et al., 2012).

Epoxide hydrolases (EHs) are a significant group of catalysts that play a key role in converting epoxide compounds to decrease their reactivity. The widely acknowledged catalytic mechanisms are illustrated in **Figure 4**. The activity of EHs within the gut microbiota was first identified as far back as 1978 using gas chromatography-mass spectrometry (Hwang and Kelsey, 1978). However, there are still few reports on their metabolic effects on natural product drugs. Jan Madacki and colleagues discovered that *Mycobacterium tuberculosis*, a pathogenic microorganism responsible for intestinal and pulmonary tuberculosis in humans, can hydrolyze 9,10-cis-epoxy stearic acid into diol *in vitro* (Madacki et al., 2021). Previous research indicates that 12,13-diHOME, a hydrolysis product of gut microbial-EHs in newborns, could serve as a potential risk marker for childhood asthma. However, the precise substrate for this compound remains unknown (Levan et al., 2019). These findings suggest that intestinal microbial-derived EHs could play a significant role in endogenous natural product metabolism. Most studies on EHs currently focus on biocatalysts. Microbiome-derived EHs, unlike those from animals and plants, do not rely on coenzymes for catalysis, making them a promising class of biocatalysts for synthesizing complex natural products (Wang et al., 2015). For example, they have been used in the synthesis of epichlorohydrin (Liang et al., 2019), chiral phenyl ethylene glycol (Jia et al., 2011), and aryl glycidyl ether (Xu et al., 2020; Zhang et al., 2020), among others.

## 3 Effects of natural product drugs on microbiologically derived hydrolases

The gut microbiome hydrolases play a crucial role in transforming the active ingredients of natural product drugs, thereby influencing the potency and bioavailability of these drugs (Zhang et al., 2018). This relationship is bidirectional, as some natural product drugs can target GMDH to modulate metabolic pathways or counteract drug effects. For instance, primary BAs have been found to inhibit microbiota-derived BSH from bacteria like *Staphylococcus, Balantidium, Pneumococcus*, and *Enterococcus (* Ridlon et al., 2016). Recent research indicates that BAs can induce DNA damage by disrupting RNA secondary structure in intestinal microbiota, impacting the levels of bile salt hydrolase. This mechanism may be crucial for maintaining bile acid pool homeostasis in the body (Watanabe et al., 2017; Bustos et al., 2018). In addition, certain natural products can also inhibit glycoside hydrolases from gut microbiota. For example, compounds like diterpenoids and linolenic acid from *Rubidium strobili* have demonstrated inhibition of glucosidase in *E. coli* (Ganzon et al., 2022; Yu et al., 2022).. Weng and colleagues conducted a comparative analysis of over 30 flavonoids to assess their ability to inhibit glycosidases derived from gut microbiota *in vitro*. The findings indicated that scutellarein, luteolin, baicalein, quercetin, and scutellarin exhibited significant inhibitory effects on glycoside hydrolases (Weng et al., 2017). The interaction between natural products and GMDH may exhibit dynamic relationships.

## 4 Interactions between hydrolases and natural product drugs affect disease development

The co-metabolism of drugs by the microbiome can lead to byproducts with different therapeutic effects than the original drug (Lai et al., 2010). Taking amygdalin, for instance, can effectively treat bronchitis and emphysema. However, when glycoside hydrolases from intestinal microorganisms transform amygdalin, it can produce hydrocyanic acid, leading to toxic reactions and worsening the condition (Jaswal and Palanivelu J, 2018). Therefore, investigating the combined regulation of hydrolases and natural drugs on disease occurrence and progression is essential. This synergistic regulation has been extensively documented for diseases such as ulcerative colitis (UC), nervous system disease, diabetes, and cardiovascular disease (Pavlović et al., 2012; Huang et al., 2019; Liu et al., 2021; Sun et al., 2023; Zhang ZW. et al., 2023). The following sections will discuss the impact of the interaction between microbiome hydrolases and natural products on various clinical diseases, aiming to elucidate the importance of this interaction for practical applications.

### 4.1 Inflammatory bowel disease

**Figure 5:**
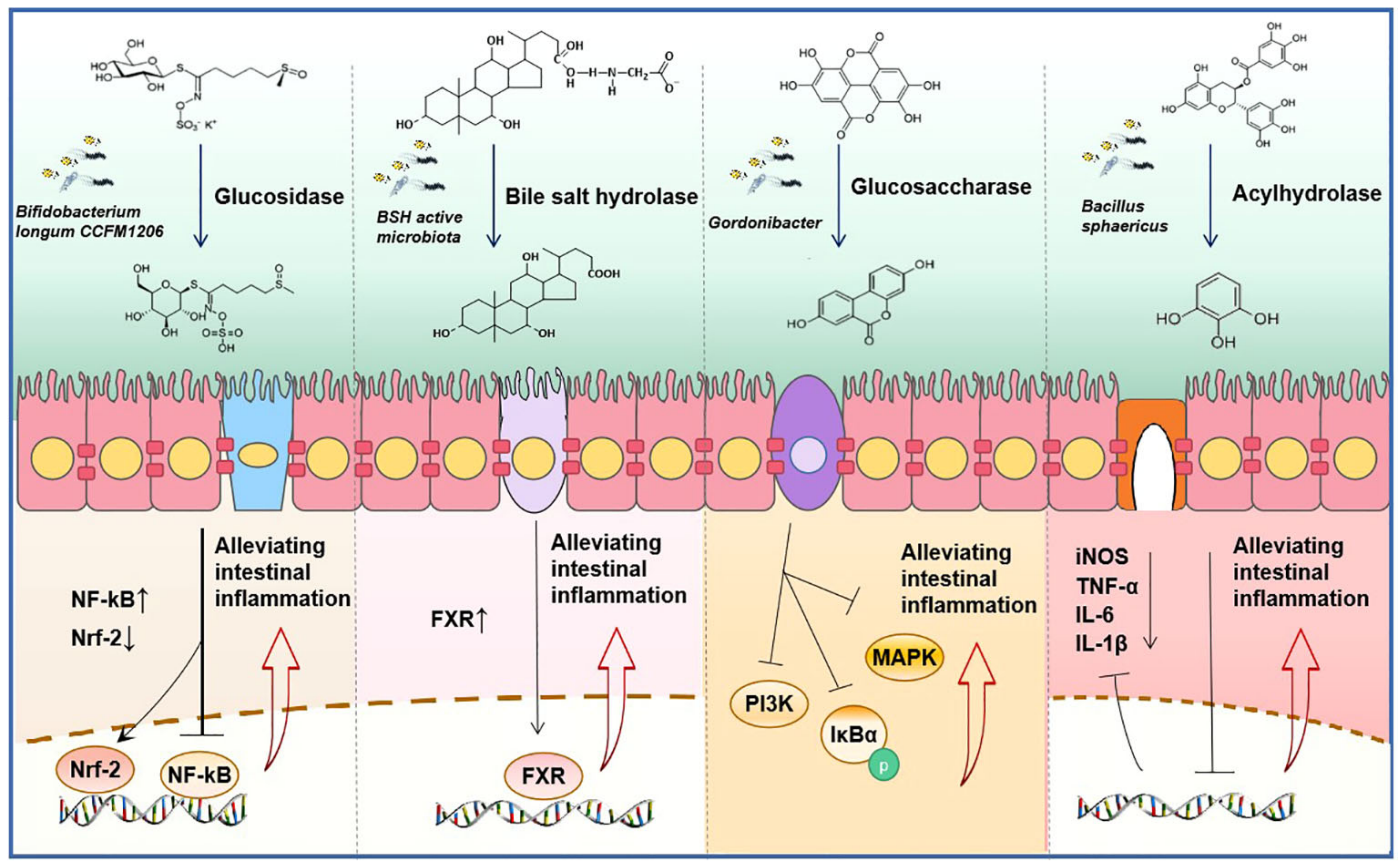
Synergistic anti-inflammatory mechanism of natural product drugs and get microbiome derived hydrolase (GMDH). From left to right, sulforaphane is hydrolyzed by glycosidases produced by *Bifidobacterium longum CCFM1206* to its active form, reducing the expression of the inflammatory mediator nuclear factor kappa-B (NF-κB) and exerting an anti-inflammatory effect (Wu J. et al., 2023). The BSH produced by microbiota converts conjugated bile acid into free bile acid, which activates the expression of farnesoid X Receptor (FXR) and exerts an anti-inflammatory effect (Gadaleta et al., 2022). Ellagic acid is metabolized to produce urolithin A by glucosidase, which inhibits the phosphorylation of IκBα and the activation of mitogen-activated protein kinase (MAPK) and phosphatidyl inositol 3-kinase (PI3K), thus displaying anti-inflammatory properties (Selma et al., 2014; Kujawska and Jodynis-Liebert, 2020; Abdelazeem et al., 2021). Acyl hydrolase derived from *Bacillus sphaericus* converts catechin gallate into pyrogallol, leading to the inhibition of the secretion of inflammatory factors (Raghuwanshi et al., 2011; López de Felipe et al., 2014).

As the gut is home to tens of thousands of microorganisms, it plays an important role in the process of intestinal diseases. Inflammatory bowel disease (IBD), comprising ulcerative colitis (UC) and Crohn’s disease (CD), is a chronic inflammatory condition that primarily impacts the gastrointestinal tract. Studies indicate that various natural products may offer relief for IBD. For instance, baicalin (Zhu et al., 2016; Wu Q. et al., 2023), *Sophora alopecuroides L* (Jia et al., 2020), fibro mushroom polysaccharid (Nguepi Tsopmejio et al., 2023), and gallic acid (Pandurangan et al., 2015) have shown promising therapeutic benefits in animal models of chemically induced colitis. We have previously explored the regulatory relationship between GMDH and natural products. However, the role of interactions between GMDH and natural product drugs in enteritis remains to be explored. The provided **Figure 5** outlines the roles of key natural products metabolized by intestinal microbiome hydrolases in enteritis, aiming to elucidate the mechanisms through which intestinal microorganisms can mitigate intestinal inflammation and lay a foundation for future research.

In summary, the regulatory role of gut microbiome hydrolases and natural products in the treatment and prevention of enteritis is a promising avenue for research. Exploring how to harness the benefits of gut microbiota and natural products in disease management is a growing area of interest.

### 4.2 Type 2 diabetes

A Mendelian randomization study conducted by Cheng demonstrated a robust association between type 2 diabetes and gastrointestinal tract diseases (Chen et al., 2023). Recent research has highlighted the importance of GMDH in the pathogenesis of type 2 diabetes. Natural products such as flavonoids, lipids, saponins, and BAs have been found to have beneficial anti-diabetic properties (Fan et al., 2023). Acarbose, a common antidiabetic medication, is subject to hydrolysis by *K. grimontii TD1* bacteria in the human intestine, leading to potential resistance to acarbose (Tian et al., 2023). The previous discussion focused on the relationship between natural product drugs and gut microbes, without delving into their specific role in diabetes. Recent evidence suggests that the microbiota-derived BSH may serve as a key factor in this interaction. Natural product drugs have been found to decrease BSH levels in the intestine by inhibiting microbiota-produced BSH enzymes. This inhibition leads to alterations in downstream signaling pathways of BAs, ultimately resulting in hypoglycemic effects. **Table 2** provides a list of natural products that have shown potential in reducing glucose levels through BSH modulation, offering novel insights and strategies for diabetes prevention and treatment.

**Table 2:** Natural products that inhibit the effects of type 2 diabetes by BSH enzymes.

Names	Microbiota	The expression of BSH	Bile acid	Signaling pathways	Pharmacodynamics
2α-OH-protoginseng diol	*Clostridiales, Gemella, Ruminiclostridium Romboutsia, Lachnospiraceae, Ruminococcaceae, Oscillibacter, Desulfovibrios ↑, Akkermansia muciniphila ↓*	Reducing the expression of BSH	T-β-MCA↑	The signaling pathways of FXR-GLP-1 are inhibited	Improving Glucose metabolism (Xie Z. et al., 2020)
Scutellaria baicalensis	*Bacteroides finegoldii and B.fragilis↓*		GUDCA and TUDCA↑	The signaling pathways of FXR-FGF19 are inhibited	Improving insulin sensitivity in type T2 diabetes (Sun et al., 2018; Hui et al., 2019)
Capsaicin	*Lactobacillus↓*		T-β-MCA↑	The signaling pathways of FXR-FGF15 are inhibited	
Epigallocatechin 3-gallate	*Akkermansia muciniphila↑*	Increasing the expression of BSH	CDCA↑	Activating the expression of FXR and TGR5	Reducing diet-induced obesity, visceral fat, and insulin resistance (Xu et al; Sheng et al., 2018; Li Y. et al., 2021)
Diammonium glycyrrhizinate	*Clostridium IV and Clostridium XlVa ↓*	Reducing the expression of BSH	Upregulate all taurine-binding bile acids	The signaling pathways of FXR-FGF15 are inhibited	
L theanine	*Lactobacillus, Streptococcus, Bacteroides, Clostridium and Enterorhabdus↓*		GCA, GLCA, GUDCA, CDCA, and CA ↑		

↑ indicates that the content is increasing, and ↓ indicates that the content is decreasing.

In addition to BSH, the aglycone produced by some flavonoids through hydrolysis also exhibits inhibitory effects on the onset and progression of diabetes. The aglycone produced by some flavonoids through hydrolysis also exhibits inhibitory effects on the onset and progression of diabetes. For instance, ginsenoside Rb1 stimulates GLP-1 secretion (Yang et al., 2021), astragaloside IV increases the levels of intestinal butyric acid (Gong et al., 2021), and resveratrol reduces the levels of inflammatory factors in the intestine (Cai et al., 2020). In conclusion, focusing on bile salt hydrolase and glycoside hydrolase among GMDH may be crucial for the development of new anti-diabetic natural product drugs.

### 4.3 Atherosclerosis

Atherosclerosis is a significant type of cardiovascular disease characterized by the formation of plaques containing necrotic cores, calcified regions, accumulated modified lipids, inflamed smooth muscle cells, endothelial cells, leukocytes, and foam cells (Fan and Watanabe, 2022). Recent studies have demonstrated that hydrolysates of flavonoid microbiota can have positive impacts on atherosclerosis. For instance, 3-hydroxyphenyl, a hydrolytic metabolite of quercetin, has been shown to effectively lower arterial blood pressure and induce vascular relaxation in rats (Najmanová et al., 2016). Ginsenosides derived from steroids have demonstrated protective effects against atherosclerosis, with this protection being attributed to intestinal microbiome hydrolases (Zhang N-N. et al., 2023). The microbiome hydrolysates Rb1, Rg2, Rg3, and compound K have been found to alleviate atherosclerosis through various pathways. For instance, Rb1 was found to inhibit neointimal hyperplasia induced by balloon infusion in rats by suppressing vascular smooth muscle cell proliferation and phenotypic switching (Yang et al., 2018). Rg1 and Rg2 play a role in boosting the levels of macrophage autophagy proteins, which are vital for sustaining macrophage function in advanced atherosclerosis (Cho et al., 2013). Additionally, compound K hinders the advancement of atherosclerosis by targeting NF-κB and JNK-MAPK pathways (Coines et al., 2019). In conclusion, the hydrolysis and conversion of ginsenosides in the intestines are crucial for unleashing the therapeutic benefits of ginsenosides and easing atherosclerosis within the body.

## 5 Limitations of research on gut microbiome-derived enzymes

The development of intestinal microbial-derived enzymes faces three main challenges. Firstly, pinpointing a specific intestinal microorganism during the metabolism of natural product drugs is challenging. Researchers commonly employ methods like fecal bacterial transplantation, antibiotic treatment, and 16S rRNA analysis to study the intestinal microbial populations involved in drug metabolism (Li Q. et al., 2021; Zeng et al., 2023). However, these methods have limitations due to inter-individual variability and variances in the gastrointestinal tract between human and animal models (Yu Y. et al., 2023). Moreover, besides bacteria, the intestine also harbors fungi, viruses, and phages. The current analysis methods primarily focus on bacteria, neglecting the roles of other microorganisms in drug metabolism (Shao et al., 2023). The advancement of high-throughput single-cell genome sequencing technology holds promise for enhancing the resolution of intestinal microorganisms (Zheng et al., 2022). The second challenge involves incubating differential strains *in vitro*. Bacteria can only be studied and identified through *in vitro* culturing, yet isolating specific groups from the extensive intestinal microbiome proves challenging. The microbiome-derived metabolism screening platform, created by Bahar Javdan, enables high-throughput screening of bacterial groups related to natural product metabolism. However, there are limitations to the strains that can be cultured *in vitro* (Javdan et al., 2020). Utilizing culturomics methods may enable the isolation of a greater diversity of bacteria in the future, potentially offering a solution to this technical challenge (Chang et al., 2019). A third challenge is the identification of key gut microbe-derived enzymes, as discrepancies persist between enzymes found in human gut microbes and data from gut microbial genomes. Approximately 40% of protein sequences lack functional annotation, making it difficult to link many enzyme sequences to specific functions (Almeida et al., 2021). Moreover, the conservation of similar gene sequences often leads to misannotation and over-prediction of enzyme molecular functions in public databases, hindering the identification of derivative enzymes in the gut microbiome (Schnoes et al., 2013; Jia et al., 2017). The advancement of bioinformatics and computer-assisted techniques can be valuable tools in overcoming this challenge (Sharma et al., 2017; Guthrie et al., 2019; Malwe et al., 2023). In summary, the advancement of single-cell genome sequencing technology, culture-omics technology, and bioinformatics analysis platforms holds promise for enhancing our comprehension of microbial drug metabolism in the future.

## 6 Conclusion

Although natural products are often valuable as lead compounds, they are seldom directly applicable in clinical settings. The bioactivation and potential health benefits of many natural products, such as flavonoids, alkaloids, and lignin, heavily rely on gut microbes acting as substrate processing plants (Singh et al., 2020).To enhance their clinical utility, it is imperative to modify the structure of natural products. Oral natural product drugs come into direct contact with the intestinal microbiota. Structural alterations of drugs by hydrolases derived from the intestinal microbiota are a crucial strategy to enhance drug efficacy, refine chemical structure, and mitigate adverse reactions (Zhang et al., 2018; Weersma et al., 2020; Xie Y. et al., 2020). Individual variations in the intestinal microbiome may account for differences in the bioavailability of certain natural product drugs among various populations, offering insights for personalized treatment approaches. The impact of natural products on microbiome hydrolase suggests a need for increased vigilance regarding potential drug interactions when combining natural product drugs. Certain drugs may interact with clinical drugs that undergo enterohepatic circulation via microbiome hydrolase-mediated mechanisms, highlighting the importance of studying combinations of natural product drugs and traditional Chinese medicine prescriptions. For instance, antibiotics like cefalexin, tetracycline, and erythromycin have been shown to notably alter the oral pharmacokinetic properties of baicalin (Kang et al., 2014). In studies, it has been found that low concentrations of clove water extract can competitively inhibit the activity of the β-D-glucuronide enzyme from *Escherichia coli* by producing the hydrolyzed product urolitin M5 (Bai et al., 2021). Additionally, amentoflavone was found to strongly inhibit the hydrolysis of 6, 8-dichloro-7-hydroxy-9,9-dimethylacridin by β-D-glucuronide (Tian et al., 2021). It is crucial to consider gut microbial hydrolases when investigating the interactions between oral natural products and drugs. All in all, the interplay of GMDH with natural product drugs significantly impacts host health, disease progression, and therapeutic outcomes, offering valuable insights into disease mechanisms and potential drug targets.

## Author contributions

JH: Writing – original draft. XL: Writing – review & editing. JZ: Writing – review & editing. RW: Writing – review & editing. XC: Supervision, Writing – original draft. GL: Funding acquisition, Writing – review & editing.
